# Cyclodextrin-Nanosponge-Loaded Cyclo-Oxygenase-2 Inhibitor-Based Topical Gel for Treatment of Psoriatic Arthritis: Formulation Design, Development, and *In vitro* Evaluations

**DOI:** 10.3390/ph17121598

**Published:** 2024-11-27

**Authors:** Umme Hani, Sharanya Paramshetti, Mohit Angolkar, Wajan Khalid Alqathanin, Reema Saeed Alghaseb, Saja Mohammed Al Asmari, Alhanouf A. Alsaab, Farhat Fatima, Riyaz Ali M. Osmani, Ravi Gundawar

**Affiliations:** 1 Department of Pharmaceutics, College of Pharmacy, King Khalid University, Abha 62529, Saudi Arabia; uahmed@kku.edu.sa; 2 Department of Pharmaceutics, JSS College of Pharmacy, JSS Academy of Higher Education and Research (JSS AHER), Mysuru 570015, Karnataka, India; paramshettisharanya@gmail.com (S.P.); mohitangolkar11@gmail.com (M.A.); 3 Department of Doctor of Pharmacy, College of Pharmacy, King Khalid University, Abha 62529, Saudi Arabia; wajankh0@gmail.com (W.K.A.); reemaghaseb@gmail.com (R.S.A.); sajamuhammed09@gmail.com (S.M.A.A.); 4 Pharmacist at Abha International Private Hospital, Abha 62521, Saudi Arabia; h99h201@gmail.com; 5 Department of Pharmaceutics, College of Pharmacy, Prince Sattam bin Abdulaziz University, Al-Kharj 11942, Saudi Arabia; f.soherwardi@psau.edu.sa; 6 Department of Pharmaceutical Quality Assurance, Manipal College of Pharmaceutical Sciences, Manipal Academy of Higher Education (MAHE), Manipal 576104, Karnataka, India; gundawar.ravi@manipal.edu

**Keywords:** cyclodextrin, nanosponge, solubility, controlled release, solubility enhancement, psoriatic arthritis, badam gum, gels, topical delivery

## Abstract

**Background:** Psoriatic arthritis (PsA), a chronic inflammatory disease, mainly affects the joints, with approximately 30% of psoriasis patients eventually developing PsA. Characterized by both innate and adaptive immune responses, PsA poses significant challenges for effective treatment. Recent advances in drug delivery systems have sparked interest in developing novel formulations to improve therapeutic outcomes. The current research focuses on the development and evaluation of a nanosponge-loaded, cyclo-oxygenase-2 (COX-2) inhibitor-based topical gel for the treatment of PsA. **Methods:** Nanosponges (NSs) were prepared by using beta-cyclodextrin as a polymer and dimethyl carbonate (DMC) as a crosslinker by melting, and gels were prepared by employing carbopol and badam gum as polymers. **Results:** Solubility studies confirmed that the prepared nanosponges were highly soluble. FT-IR studies confirmed the formation of hydrogen bonds between lumiracoxib and beta-cyclodextrin. SEM confirmed that the prepared formulations were roughly spherical and porous in nature. The average particle size was 190.5 ± 0.02 nm, with a zeta potential of −18.9 mv. XRD studies showed that the crystallinity of lumiracoxib decreased after encapsulation, which helped to increase its solubility. The optimized nanosponges (NS2) were incorporated in an optimized gel (FG10) to formulate a nanosponge-loaded topical gel. The optimized gel formulation exhibited a homogeneous consistency, with a pH of 6.8 and a viscosity of 1.15 PaS, indicating its suitability for topical application and stability. The *in vitro* diffusion studies for the topical gel showed drug release of 82.32% in 24 h. The optimized formulation demonstrated significant antipsoriatic activity, as confirmed through cytotoxicity studies conducted on HaCaT cells. **Conclusions:** On the basis of the findings, it can be concluded that the prepared nanosponge-loaded topical gel formulation presents a promising solution for the effective management of PsA, offering enhanced drug solubility, sustained release, and improved therapeutic potential.

## 1. Introduction

Psoriasis is known to be an immune-mediated, genetic papulosquamous skin disorder. It is the most prevalent chronic autoimmune condition in the US. It is a skin condition that affects approximately 3% of individuals in the US [1]. Pro-inflammatory cytokines and growth factors are released by the immune system, leading to the accumulation and formation of red patches on different areas of the body [2,3]. About 30% of people with psoriasis develop inflammatory arthritis, a condition in which the inflammation spreads to the joints and enthuses [3,4,5,6]. Patients with psoriatic arthritis (PsA) can experience swelling, stiffness, and joint pain in any part of the body [7,8]. In PsA, inflammation of the skin and joints is caused by the release of cytokines by immune cells, which then act on healthy cells and tissues. The activation of cellular pathways leading to skin and joint disease is triggered by the interplay of genetic and environmental factors [9,10].

PsA was long ignored as a minor condition with no long-term effects, and treatment was therefore regarded as unnecessary [11]. For effective treatment of PsA, novel drug delivery systems such as nanoparticles, topical gels, nanoemulsions, niosomes, hydrogels, solid lipid nanoparticles (SLNPs), liposomes, microemulsions, and ethosomes are mostly used today [12,13,14]. Among them, topical gels are a very attractive drug delivery system. These are semisolid systems that contain either a liquid surrounded by large organic molecules or a suspension of small inorganic particles [15]. In terms of drug loading, when topical gels are compared with other nanoparticles, they are the most suitable carriers. Following administration, the topical gels carry the payload safely, move within the cells, and release the contents where they are needed *in vivo* [16].

Prostaglandins are molecules that play a significant role in the inflammation linked to arthritic pain, fever, edema, and tenderness. A selective cyclo-oxygenase-2 (COX-2) inhibitor, lumiracoxib (LXB), has been suggested as oral therapy for the treatment of arthritis and osteoarthritis. This anti-inflammatory agent is also used to treat psoriatic arthritis. However, prolonged oral LXB use results in significant gastrointestinal adverse effects. Also, lumiracoxib has poor aqueous solubility. In this study, the potential of nanosponges (NSs) for the delivery of lumiracoxib was investigated in order to decrease the adverse effects associated with oral administration of the drug, improve its solubility and bioavailability, and offer sustained release [17,18].

Nanosponges are an innovative method that allows regulated drug delivery for topical usage [19,20,21]. NSs represent a burgeoning technology for delivering drugs topically and augmenting the efficacy of topically administered drugs. Various drugs can be integrated into nanosponges, which are minute sponge-like structures comparable in size to a virus. These diminutive sponges can navigate through the body until they reach the designated target area, where they adhere to the surface and initiate a controlled and predictable release of the drug [22,23,24]. The application of nanosponges in controlled drug delivery has positioned them as one of the most promising domains in life science [25,26].

The trapping of drugs by nanosponge technology is believed to minimize adverse effects, improve stability, enhance elegance, and offer increased formulation flexibility. These are non-mutagenic, non-allergenic, non-toxic, and non-irritating [27]. Cyclodextrins (CDs) are the most preferred polymer in the preparation of nanosponges, as they have a greater capacity to enhance the solubility of drugs that are poorly soluble [28]. Cyclodextrins are biomaterials at the nanometric scale, showcasing a direct correlation between their supramolecular properties and molecular state. Enzymatic activity on hydrolyzed starch produces these cyclic glucopyranose oligomers. The three most prevalent natural cyclodextrins containing six, seven, and eight glucopyranose units are α, β, and γ, respectively [29].

Recent advancements include NSs, composed of microscopic particles with nanometer-sized cavities. These innovative nano-sized colloidal carriers, tailored for drug delivery, demonstrate the ability to solubilize water-insoluble drugs, prolong release, and enhance drug bioavailability by modifying pharmacokinetic parameters. CDs play a crucial role in forming inclusion complexes with drug molecules, enhancing solubility, masking undesirable characteristics, and improving photostability or aqueous stability in pharmaceutical applications. CD-based NSs, formed through hyper-crosslinking of different CD molecules with crosslinkers like carbonyl or carboxylate compounds, exhibit the properties of crosslinked polymers, including chemical absorption or inclusion, swelling, and efficient release of active ingredients. NSs surpass other nanocarriers in terms of high drug-loading capacity and offer promising solutions for addressing solubility, bioavailability, controlled release, and stability concerns across various therapeutic agents. Furthermore, NS formulations can be seamlessly integrated into conventional dosage forms like gels, ointments, lotions, creams, and powders, expanding their versatile applications [30,31]. Thus, the aim of the current study was to develop a cyclodextrin-nanosponge-based topical gel to achieve a sustained drug release profile for the encapsulated drug, and to enhance therapeutic efficacy. By improving drug solubility and ensuring sustained release, this formulation seeks to provide a more effective and convenient treatment option for psoriatic arthritis, ultimately facilitating better therapeutic outcomes and management.

## 2. Results and Discussion

### 2.1. Solubility Efficiency

The prepared nanosponges had higher solubility than the pure compound in the phosphate buffer (pH 7.4). The solubility of lumiracoxib was enhanced by 28–36 times in the prepared nanosponge formulation. This is because the drug’s solubility is enhanced by β-cyclodextrin. The increase in LXB’s solubility was attributed to its entrapment within the matrix and the formation of inclusion complexes. The primary determinant of the formation of inclusion complexes was the level of crosslinking between the nanosponges and LXB. A greater degree of crosslinking was achieved between the nanosponges and LXB, corresponding to increased inclusion complexation and drug entrapment/loading, ultimately resulting in improved solubility. Furthermore, the enhanced solubilization of NSs can be attributed to the potential masking of LXB’s hydrophobic groups, reduced crystallinity, and improved wetting behavior, as previously reported in earlier studies [32,33,34]. The solubility of the formulated NS particles ranged between 363.74 ± 0.016 and 476.24 ± 0.017 μg/mL (Table 1). NS2 was considered to be the optimal formulation, since it showed the highest solubility.

### 2.2. Entrapment Efficiency

The EE of the LXB nanosponge formulations is given in Table 2. Encapsulation efficiency was determined on the basis of the amount of drug entrapped in the β-cyclodextrin [26]. The EE of the nanosponge formulations was found to be in the range of 64.24% to 72.38%. The variation in LXB entrapment revealed that their degree of crosslinking influenced the ability of the nanosponges to form inclusion complexes. The highest loading efficiency was found in the NS2 formulation. The highest drug loading in the NS2 formulation (72.38%), in comparison to the other two formulations, was likely due to more extensive crosslinking between β-cyclodextrin (β-CD) and dimethyl carbonate, which facilitates the encapsulation of LXB within the internal structure of the NSs. The differences in LXB entrapment indicate that the degree of crosslinking significantly impacts the complexation capacity of the nanosponges. In the case of NS1, the lower amount of crosslinker resulted in an incomplete network, with fewer cyclodextrin crosslinking points, reducing the available sites for LXB complexation. As a result, less LXB was encapsulated. In contrast, the higher crosslinker concentration in NS2 allowed for more extensive β-CD crosslinking, which enhanced the interaction between LXB and the cavities of β-CD, leading to improved drug entrapment, as previously reported by Ansari et al. [35] and Shende et al. [36]. NS2 was chosen as the optimized formulation for further studies because of its excellent solubilization and entrapment efficiency. The encapsulation studies confirmed that dimethyl carbonate is an effective crosslinking agent for the preparation of NSs.

### 2.3. Characterization of Lumiracoxib β-Cyclodextrin Nanosponges

#### 2.3.1. FT-IR Studies

**Figure 1:**
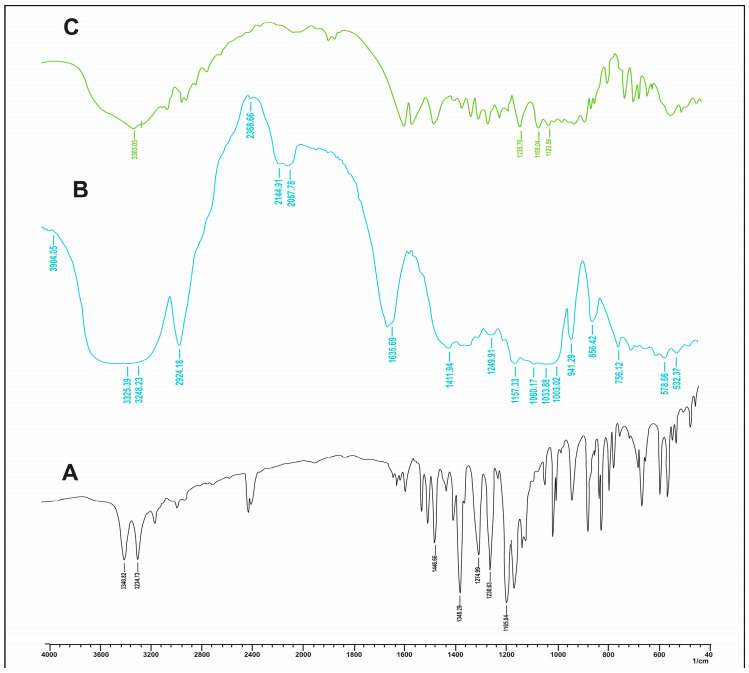
FT-IR spectra of (**A**) lumiracoxib, (**B**) β-cyclodextrin, and (**C**) lumiracoxib-loaded β-CD NSs (NS2).

FT-IR studies anticipate potential interactions occurring between the drug and the formulation. The FT-IR spectra of lumiracoxib, β-CD, and NS2 are depicted in Figure 1. As shown in Figure 1A, the characteristic peaks of lumiracoxib were observed as S=O at 1165.04 cm^−1^, C-F at 1135 cm^−1^, C-C vibrations at 1232.15, C-N stretching at 1275 cm^−1^ and 1349 cm^−1^, and N-H stretching at 3235 cm^−1^ and 3341 cm^−1^. For the β-cyclodextrin, characteristic peaks were observed as O-H stretching of intracavity H_2_O at 3530.23 cm^−1^, O-H stretching of primary alcohol groups at 3370.12 cm^−1^, and O-H stretching of secondary alcohol groups at 3245.28 cm^−1^. These were the characteristic peaks that were responsible for complex formation, as depicted in Figure 1B.

The overlaid spectra of lumiracoxib and the NS formulation are represented in Figure 1C. The nanosponge spectra show that there was a significant change in the fingerprint region. There was a broadening of the peak at the functional group region, which corresponds to the O-H primary alcohol group peak of cyclodextrin, which underwent a downshift at 3306.03 cm^−1^, confirming the formation of a bond between lumiracoxib and beta-cyclodextrin. This shift signifies the establishment of a new molecular interaction, confirming the encapsulation of lumiracoxib within the β-cyclodextrin framework.

#### 2.3.2. DSC Studies

**Figure 2:**
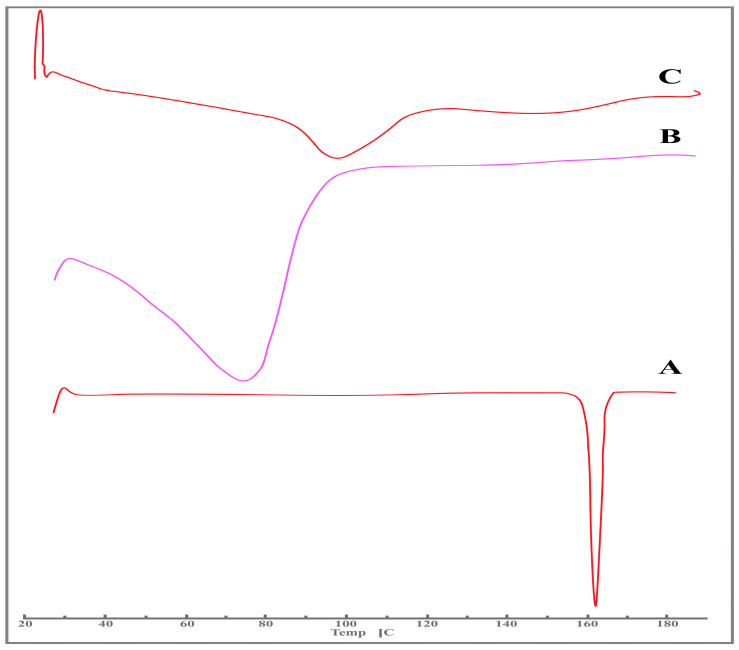
DSC thermograms of (**A**) lumiracoxib, (**B**) β-cyclodextrin, and (**C**) lumiracoxib-loaded β-CD NSs (NS2).

The DSC thermograms of the pure drug, β-CD, and NS2 are depicted in Figure 2. Figure 2A depicts the DSC analysis of pure lumiracoxib, demonstrating a sharp linear endothermic peak at a temperature of 162.10 °C, which is suggestive of its melting point. The DSC analysis of β-CD is shown in Figure 2B. This endothermic peak was also identified in relation to the formulation of NS2 (represented in Figure 2C), corresponding to the melting temperature of the pure drug. The DSC thermograms demonstrated that the excipients and the drug differed significantly. The thermograms obtained indicated that the encapsulation of LXB into NSs led to a discernible shift in its melting point. This shift was accompanied by a change in the glass transition temperature, indicating a transition from the crystalline form of lumiracoxib to an amorphous state. Such transformation strongly suggests the formation of a complex between LXB and β-CD, as evidenced by the altered thermal properties observed in the thermograms.

#### 2.3.3. XRD Studies

**Figure 3:**
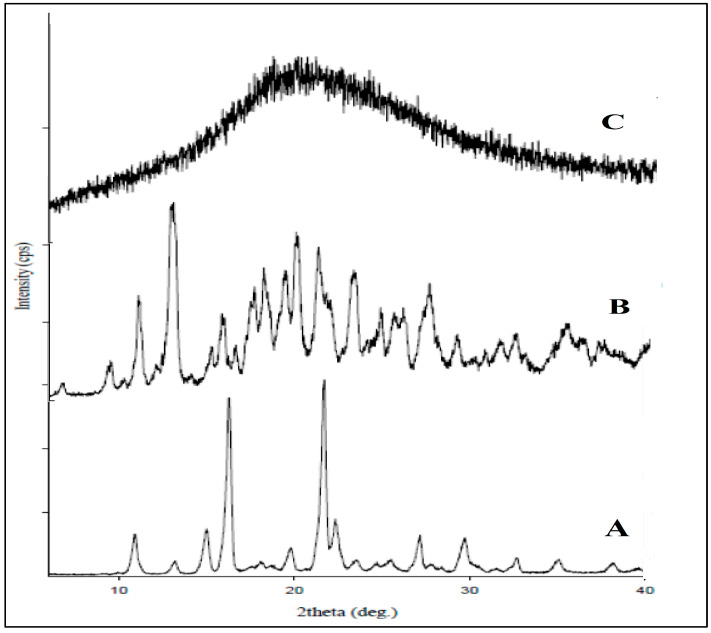
X-ray diffractograms: (**A**) lumiracoxib, (**B**) β-cyclodextrin, and (**C**) lumiracoxib loaded β-CD NSs (NS2).

The crystallinity of the formulation was assessed using XRD analysis. The XRD spectra of lumiracoxib, β–CD, and the optimized formulation NS2 are depicted in Figure 3. The lumiracoxib and β-CD exhibit intense, sharp diffraction peaks indicating their crystalline nature, as represented in Figure 3A,B respectively. The XRD pattern of the lumiracoxib-loaded β-CD NSs (Figure 3C) shows the absence of sharp diffraction peaks, indicating a reduction in the peak intensities, confirming the conversion of the drug into its amorphous form. The crystallinity of LXB was notably reduced following its encapsulation into NSs, resulting in its transition to an amorphous state. These observations offer strong evidence that the drug’s interaction and complexation were driven by more than just the mechanical mixing of the drug and the excipients. These findings are consistent with the results obtained from the FT-IR and DSC analyses, further supporting the conclusion that a chemical interaction between the constituents occurred during the formulation process.

#### 2.3.4. Particle Size (PS) and Zeta Potential Analysis

**Figure 4:**
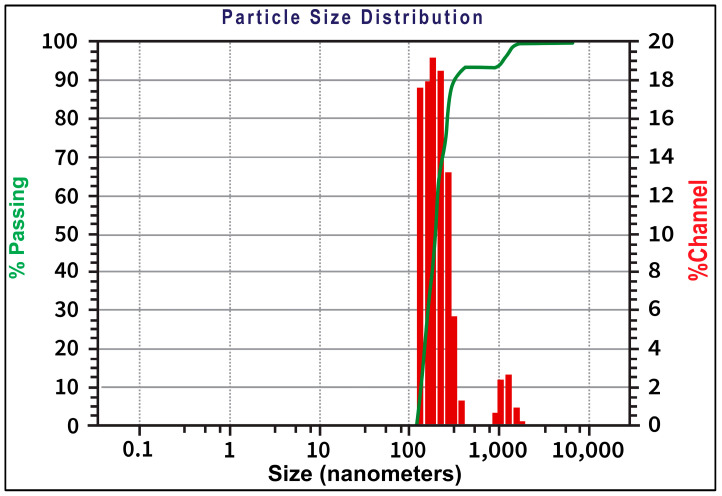
Particle size distribution for NS2 formulation.

The mean PS of the optimized formulation NS2 was measured by DLS with the help of a Zetasizer. As depicted in Figure 4, the average diameter of the optimized formulation NS2 was around 190.5 nm. Zeta potential is a measure of the electrostatic potential at the slipping plane of a particle’s surface. It helps predict the potential for particles to repel or attract each other, influencing their aggregation or dispersion behavior. In the context of nanosuspensions, which are colloidal systems consisting of finely dispersed nanoparticles in a liquid medium, zeta potential is particularly important for maintaining stability. Typically, a zeta potential of around +30 mV or −30 mV is considered good enough to overcome any possibility of particle aggregation in the nanosuspension, thereby providing stability to the nanoformulation. The optimized formulation was found to have a zeta potential of −18.9 mv, indicating that the particles are moderately stable. It is important to note that while zeta potential provides valuable insights into formulation stability, it is not the sole factor determining stability. Other factors, such as particle size, surface chemistry, and interactions between particles and the surrounding medium, also contribute to the overall stability of a nanosuspension.

#### 2.3.5. Scanning Electron Microscopy

**Figure 5:**
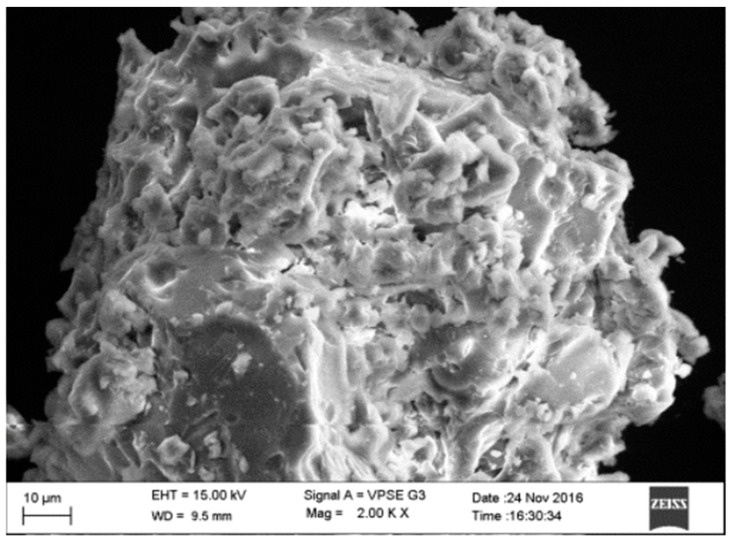
Surface morphology of optimized nanosponge (NS2).

The morphology and size of the NSs were examined using scanning electron microscopy. The optimized nanosponge (NS2) was found to be porous in nature and roughly irregular in shape. The SEM micrograph displayed in Figure 5 reveals the surface morphology of the NS, showcasing numerous fine surface voids. These voids are likely the consequence of solvent diffusion during the formation process. Notably, no residual LXB was observed on the NS surface. This absence suggests that the nanosponge matrix is primarily formed by the inclusion of LXB within β-CD, rather than by the presence of separate LXB crystals.

### 2.4. In vitro Drug Diffusion Studies

**Figure 6:**
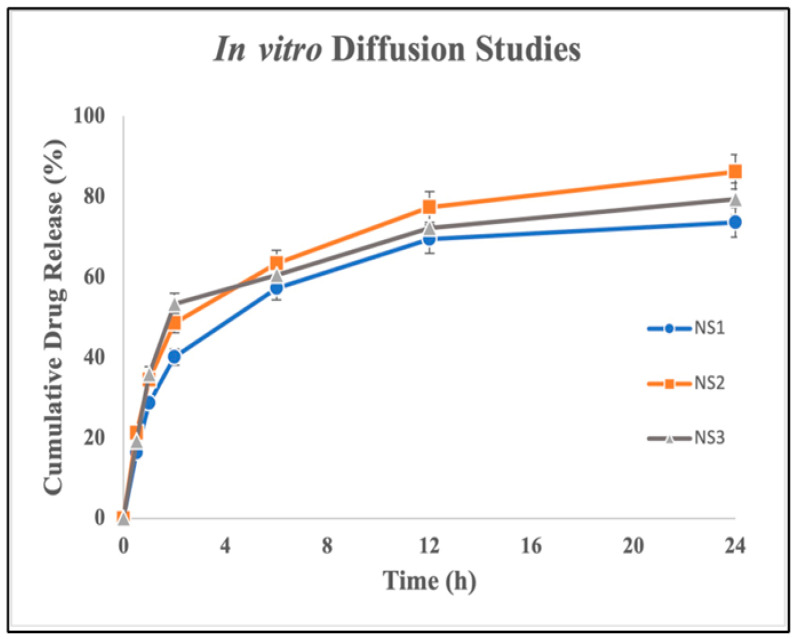
Graph of *in vitro* diffusion studies for formulations NS1 to NS3. Each value is expressed as the mean ± SD, with experiments conducted in triplicate (n = 3).

*In vitro* drug diffusion tests were conducted for the prepared nanosponges. From the obtained *in vitro* data, a graph was plotted against the percentage of cumulative drug release vs. time. The obtained data are tabulated in Table 3. Figure 6 depicts plots the percentage of cumulative drug release for the different formulations. Notably, the NS2 formulation, characterized by a polymer-to-crosslinker ratio of 1:4, exhibited the highest *in vitro* release. This enhanced release can be attributed to the presence of non-inclusion complexes in NS1, stemming from inadequate nanopores or nanochannels. Conversely, in the NS3 formulation, lumiracoxib’s incapacity to penetrate the larger network of channels and pores contributed to the observed release pattern. Due to the presence of cyclodextrins in the NS structure, the release of lumiracoxib *in vitro* was delayed.

### 2.5. Optimization of Gel

The NS-loaded gel was prepared to ensure a controlled and prolonged release of lumiracoxib. Employing a comprehensive full factorial design (Table 4, nine distinct formulations of lumiracoxib–CD NS-based gel, denoted as FG1 to FG9, were developed. This involved altering the concentrations of two independent variables: carbopol and badam gum.

**Figure 7:**
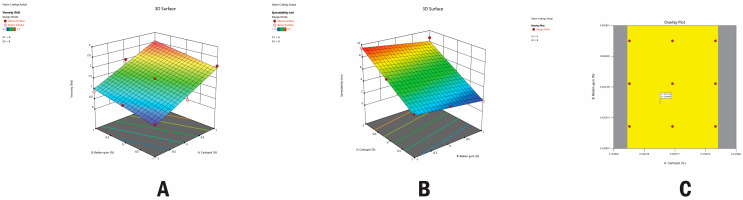
Three-dimensional (3D) response surface plots of (**A**) viscosity (PsA) and (**B**) spreadability (cm). (**C**) Overlay plot of optimized gel formulation.

The resultant response values, depicted in Table 4 as R1 (viscosity, in PaS) and R2 (spreadability, in cm), underwent a sophisticated analysis through multiple regressions. These analyses resulted in the derivation of polynomial equations, wherein the coefficient values elucidated the impact of individual variable alterations. To enhance comprehension, three-dimensional response surface graphs were plotted, as depicted in Figure 7. The figure illustrates that increasing the concentrations of carbopol and badam gum leads to higher viscosity in the formulation, resulting in a thicker texture that is more resistant to flow. However, this increase in viscosity is accompanied by a decrease in spreadability. Thus, there is an inverse relationship: higher concentrations of these components lead to greater viscosity but reduced ease of spreading.

### 2.6. Checkpoint Analysis of Gel

For optimizing various responses with distinct targets, a meticulous multi-criteria decision-making approach was implemented, as illustrated in Figure 7C. The optimized gel formulation (FG10) was formulated by applying constraints of A = 0.27 and B = 0.45 on the responses. The concentrations of the dependent factors were meticulously determined through the design of experiments, utilizing plots that exhibited the highest desirability approaching 1.0. Subsequently, the optimized formulation, FG10, underwent preparation for a checkpoint analysis, where its viscosity (PaS) and spreadability (cm) were rigorously evaluated. The predicted and observed values (Table 5) were in close agreement, with desirability values nearing 1. This alignment between predicted and actual values underscores the precision and accuracy of the optimized formulation prepared using the 3^2^ full factorial design.

### 2.7. Characterization of Topical Gels

#### 2.7.1. pH Determination

The pH of the optimized FG10 formulation was found to be 6.8 ± 0.5. This alignment with the pH of the skin holds paramount importance in topical formulations. Deviations from the skin’s natural pH can potentially lead to skin irritation. Therefore, maintaining pH within this range is crucial for ensuring compatibility with the skin.

#### 2.7.2. Homogeneity

The homogeneity of the optimized gel formulation (FG10) was visually inspected [37,38,39,40]. It was noted that the prepared formulation exhibited clarity and was devoid of any visible aggregates. These findings serve as confirmation of the uniformity and consistency achieved in the gel preparation.

#### 2.7.3. Viscosity

Viscosity is one of the most critical rheological parameters because it defines a material’s resistance to flow. The viscosity increases with an increase in the concentration of carbopol and badam gum. The viscosity of the FG10 formulation was found to be 1.15 PaS ± 0.05.

#### 2.7.4. Spreadability Studies

Spreadability is a critical attribute of semisolid dosage forms, influencing patient adherence and ease of applicability. It is worth noting that viscosity and spreadability share an inverse relationship: higher viscosity tends to decrease spreadability. In our study, the optimized gel formulation (FG10) demonstrated a spreadability of 7.1 ± 003 cm. The formulation exhibited favorable spreadability characteristics, ensuring ease of application and enhancing the overall user experience.

#### 2.7.5. Extrudability

Viscosity plays a pivotal role in the formulation of semisolid products, as the products should be viscous enough to impede phase separation while maintaining fluidity for smooth extrusion. Low-viscosity gels may flow rapidly and easily extrude from the tube, while high-consistency gels may struggle to be dispensed. Therefore, achieving a suitable consistency is crucial for ensuring proper extrusion from the tube. The extrudability of the optimized gel formulation (FG10) was found to be good.

#### 2.7.6. Drug Content

The formulated gel was analyzed to determine its drug content. The percentage drug content in the gel formulation was 92.78 ± 0.012%. This finding indicates that the topical gel’s drug content meets satisfactory levels. Moreover, it was observed that the drug was distributed uniformly throughout the formulation, ensuring consistent dosage delivery and efficacy.

#### 2.7.7. Rheological Studies

**Figure 8:**
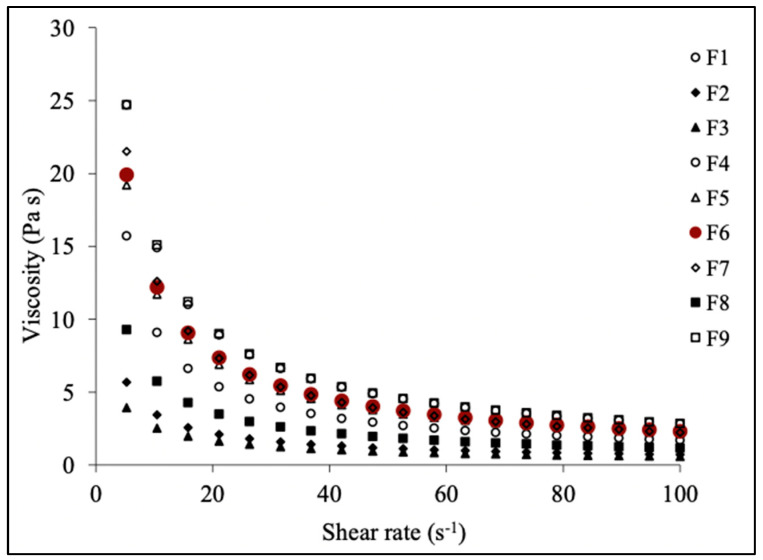
Viscosity vs. shear rate graph.

Assessing the rheological behavior of fluid formulations typically involves measuring shear stress and shear rate data. Additionally, experimental data are often represented using rheograms and empirical equations, which may consider factors such as temperature, concentration, particle size, and processing techniques. The rheological properties of topical formulations have a significant impact on both their ease of applicability or retention on the surface and spreadability. Understanding these properties helps in optimizing the formulation characteristics to ensure desired application behavior and effective retention on the skin’s surface. The rheology testing conducted on the gels revealed that all prepared formulations exhibited a non-Newtonian, pseudoplastic, thixotropic behavior at a temperature of 25 °C. This rheological profile suggests that the gels would spread effectively when applied topically. These findings are visually represented in Figure 8, offering a comprehensive understanding of the rheological properties of the gel formulations.

#### 2.7.8. *In vitro* Drug Diffusion Studies

**Figure 9:**
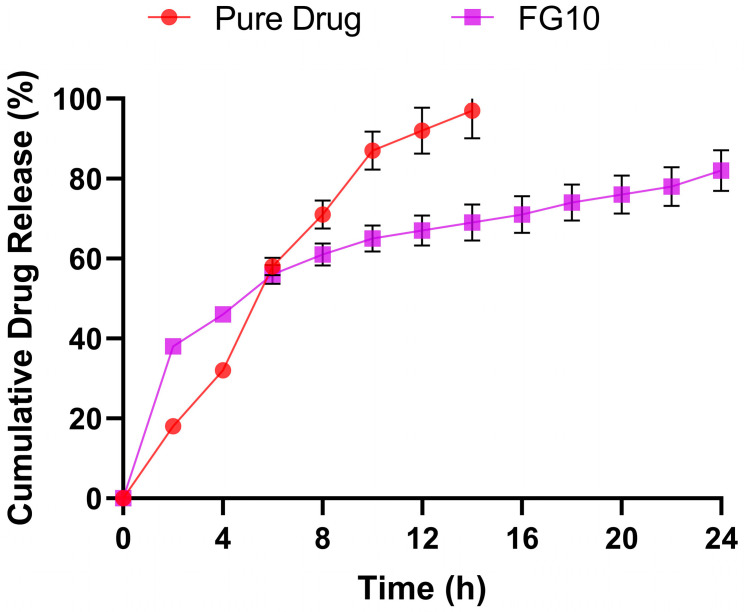
*In vitro* diffusion studies for topical gel and pure drug. Each value is expressed as the mean ± SD, with experiments conducted in triplicate (n = 3).

The topical gel formulated using the NS2 formulation showed effective *in vitro* drug release characteristics. A comparison with the pure drug revealed that while the pure drug exhibited 94.56% drug release for 14 h, the topical gel formulation displayed a higher and sustained release, lasting until 24 h and reaching a maximum of 82.56%. This extension in release duration can be attributed to the prolonged drug release profile of the NS2 nanosponge formulation, as illustrated in Figure 9. The sustained release capability of the nanosponge formulation contributes to the prolonged and controlled release of the drug from the topical gel, enhancing its therapeutic efficacy and potentially improving patient compliance.

### 2.8. Mathematical Model Fitting

The results of *in vitro* drug release tests of a topical gel formulation containing nanosponges were subjected to analysis using various mathematical models. The objective was to identify the most suitable or best-fitting model that accurately represents the release behavior of LXB from the optimized formulation. Table 6 summarizes the best-fitting models for all formulations, determined based on the highest regression coefficients (R^2^).

According to the data, the Peppas model emerged as the best-fitting model for the topical gel formulation. The obtained values of n were consistently greater than 1 when fitting the data from the formulations’ *in vitro* release tests to both the Peppas and Korsmeyer equations. Among the models examined, the Peppas model proved to be the most appropriate for describing the behavior of the FG7 topical gel formulation. This model consistently exhibited higher regression coefficients compared to all other models, indicating its superior fit to the data. The Peppas model explains the drug release mechanisms from polymeric nanoparticle-based dosage forms, such as hydrogels, particularly when multiple kinetic processes are involved. When the release exponent n falls between 0.5 and 1, it indicates non-Fickian diffusion. In this scenario, the drug is initially released from the nanosponges into the gel matrix, and then it diffuses through the gel matrix onto the skin in a controlled manner, combining both diffusion and polymer relaxation processes [41,42].

### 2.9. Cytotoxicity Profile on L929 and HaCaT Cell Lines

**Figure 10:**
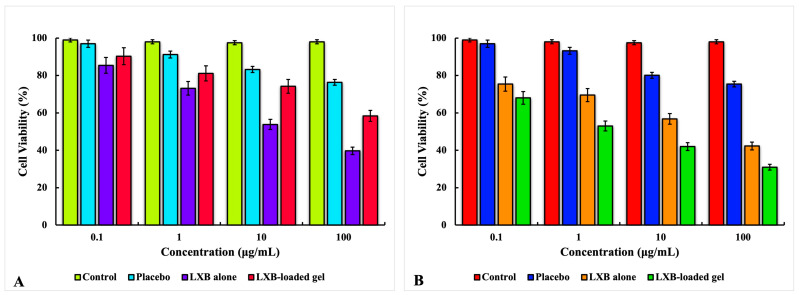
Cytotoxicity study performed using MTT assay for (**A**) L929 and (**B**) HaCaT cell lines. Each value is expressed as the mean ± SD, with experiments conducted in triplicate (n = 3).

Figure 10 illustrates the cytotoxicity of the control, placebo, free LXB, and optimized gel formulation (FG10) at concentrations ranging from 0.1 to 100 µg/mL in the HaCaT and L929 cell lines, as assessed by the MTT assay. The viability of both cell lines remained relatively stable when treated with plain NSs. In the case of L929 cells, cell viability decreased in a concentration-dependent manner for free LXB and the optimized gel formulation (FG10). However, the encapsulation of LXB in NSs improved cell viability compared to free LXB (Figure 10A).

For the highly proliferative HaCaT cells, LXB exhibited specific cytotoxicity, with an IC_50_ of 25.353 ± 0.386 µg/mL. This cytotoxic effect was further enhanced when LXB was encapsulated in the NSs and loaded in the gel, reducing the IC_50_ to 6.548 ± 0.356 µg/mL (Figure 10B). The enhanced antiproliferative activity of the optimized formulation is likely due to the improved cell uptake of NSs via endocytosis, which facilitates rapid internalization into the cytoplasm and enables more effective drug release, thereby increasing interaction with its target and causing cell death.

### 2.10. In vivo Studies

- Skin irritation studies

**Figure 11:**
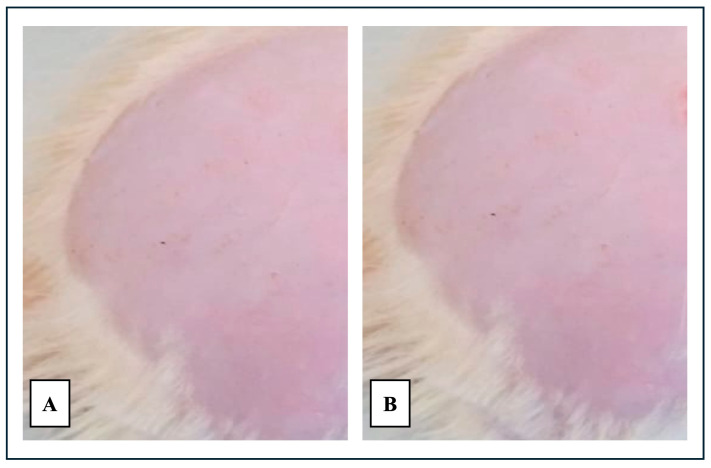
Representative images of test animal for skin irritation at (**A**) 0 h and (**B**) 72 h.

After applying the optimized NS formulation onto the skin, the presence of edema and/or erythema was assessed at 0, 24, 48, and 72 h post-application (Figure 11). The observations are summarized in Table 7. The results of the study demonstrate the compatibility and non-irritant nature of the prepared optimized FG10 formulation.

b. Skin inflammation studies

Erythema and scaling were independently evaluated using the clinical PASI score on a scale of 0 to 4, as described in the Materials and Methods section. The scoring results for the different groups are presented in Table 8.

### 2.11. Ex vivo Permeation Studies

**Figure 12:**
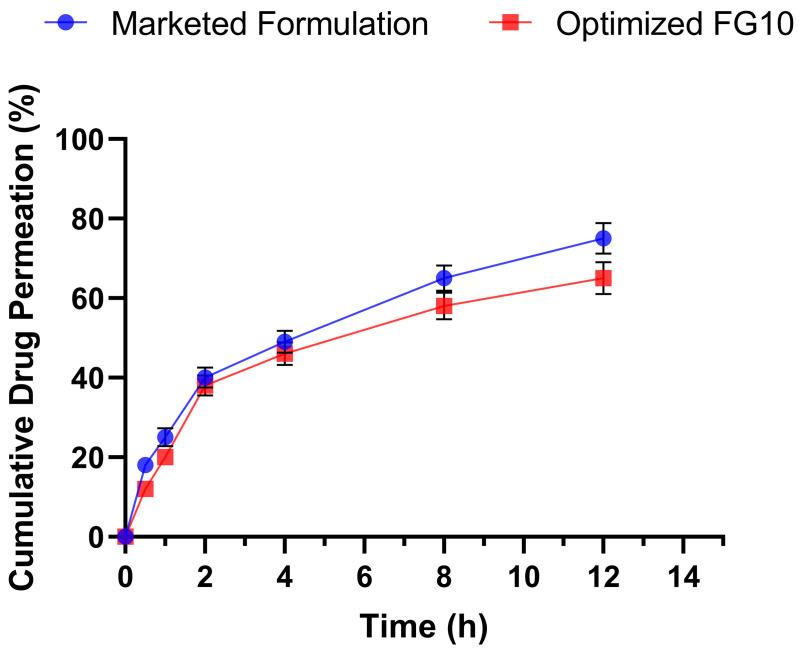
Graphical representation of *Ex vivo* permeation studies.

*Ex vivo* permeation studies demonstrated that the drug release for both of the formulations, i.e., the marketed and optimized (FG10) formulations, lasted up to 12 h. The marketed formulation showed drug release of 76.8%, whereas the FG10 formulation showed a maximum of 64.96% drug release (Figure 12). The developed nanosponge-based topical gel formulation successfully achieved sustained drug release, ensuring a prolonged therapeutic effect for improved treatment outcomes.

### 2.12. Stability Studies

Following the ICH guidelines, the stability of the optimized formulation was assessed through storage at 25 °C/60% RH for a duration of 30 days. At regular intervals, the samples were examined and assessed for any alterations in their physical attributes and drug content. Based on the data obtained (Table 9), no indications of chemical alterations or interactions within the formulations were observed throughout the study period. This stability assessment underscores the robustness and integrity of the optimized formulation under the specified storage conditions, affirming its suitability for further development and potential application.

## 3. Materials and Methods

Lumiracoxib was procured from Sigma-Aldrich Chemicals Pvt. Ltd., Bangalore, India. Carbopol 940 and dimethyl carbonate (DMC) were obtained from Loba Chemie, Mumbai, India. β-Cyclodextrin (β-CD) was purchased from Himedia, Mumbai, India. Triethanolamine and dimethyl formamide (DMF) were purchased from Merck Specialities Pvt. Ltd. Mumbai, India. Badam gum was procured from Gopala Shetty and Son’s store, Mysuru, India.

### 3.1. Synthesis of β-Cyclodextrin Nanosponges

Three different NS formulations were prepared containing β-cyclodextrin and dimethyl carbonate (crosslinking agent) at ratios of 1:2, 1:4, and 1:8. In DMF, a calculated amount of β-CD was completely dissolved. DMC was added to a round-bottomed flask (RBF) containing the above mixture. The resultant solution was reacted at 100 °C for 4 h to obtain nanosponges. The mixture was crushed in a mortar and extracted using ethanol and water to separate out unreacted DMC and impurities. The NSs were purified and then stored at 25 °C. Table 10 presents the formulation chart of the nanosponges [35].

### 3.2. Incorporation of Lumiracoxib into Nanosponges

In a beaker with 20 mL of distilled water, an accurately weighed quantity of the NS formulations was suspended. To this mixture, lumiracoxib was added in excess and subjected to sonication for 15 min. Subsequently, the mixture was left on a magnetic stirrer for 24 h. The resultant suspension was centrifuged (C-24BL, REMI, Mumbai, India) for 10 min at 2000 rpm to separate out the uncomplexed drug that accumulated as a residue beneath the colloidal supernatant [32,33].

### 3.3. Solubilization Efficiency

The solubility of the drug was assessed by adding an excessive amount of pure lumiracoxib and the nanosponge formulations to vials containing water and pH 7.4 phosphate buffer maintained at a temperature of 37 ± 0.5 °C. These vials were then shaken at 100 rpm for 24 h on an orbital shaker. The final mixture was filtered using a 0.45 μm membrane filter. The concentration of the samples was determined using a UV–visible spectrophotometer (Shimadzu, Kyoto, Japan). Each sample was analyzed in triplicate [43,44].

### 3.4. Entrapment Efficiency (EE)

To determine the EE, an accurately weighed amount of NSs equivalent to 100 mg was dissolved in pH 7.4 phosphate buffer and agitated for 10 min to break down the complexes. After filtration, 2 mL of the sample was pipetted out from the resultant solution and diluted with a 7.4 pH buffer up to 10 mL. The absorbance of the sample was measured at a wavelength of 239 nm using a UV–visible spectrophotometer (Shimadzu, Kyoto, Japan). EE can be calculated by using the following formula [43,45]:

(1) $$\mathrm{Entrapment}\;\mathrm{efficiency}\;=\;\frac{Actual\;drug\;content\;in\;nanosponges\;}{Theoretical\;drug\;content}\times 10$$

### 3.5. Fourier-Transform Infrared (FT-IR) Spectroscopic Analysis

FT-IR analysis was carried out to confirm the interaction occurring between the polymer and the drug. KBr powder was mixed with the sample, and a pressure of 4 Kg/cm^2^ was applied to form the pellets. With the help of powder diffuse reflectance, FT-IR spectra were recorded using an FTIR-8400S spectrometer (Shimadzu, Kyoto, Japan).

### 3.6. Differential Scanning Calorimetry (DSC)

DSC (DSC-60, Shimadzu, Kyoto, Japan) was carried out for the drug and nanosponge formulations. Highly pure α-alumina discs were used as the reference to perform the calorimetric measurements. The dynamic scans were conducted with a heating rate of 20 °C per minute in a nitrogen atmosphere. The energy was measured in J/Kcal.

### 3.7. X-Ray Diffraction (XRD)

MiniFlex 2 was utilized as an analyzer, while a Rigaku MiniFlex 2 Desktop X-ray Diffractometer, Rigaku, Tokyo, Japan was used to record the XRD patterns of the samples. The “Treor90” program provided by Mysore University was utilized to determine the data.

### 3.8. Particle Size and Zeta Potential Analysis

The dynamic light scattering (DLS) method was employed to determine the mean particle size distribution and charge on the surface of the resulting nanosponges using a Malvern particle size analyzer, Malvern Panalytical, Malvern, UK. The analysis was carried out using a clear zeta cell (disposable), with water as the dispersant, which had a viscosity (cP) of 0.898, a refractive index (RI) of 1.330, and the temperature maintained at 25 °C. The sample was tested in triplicate in order to minimize errors.

### 3.9. Scanning Electron Microscopy (SEM)

SEM (EVOLS15, Carl Zeiss, AG, Oberkochen, Germany) was employed to determine the surface morphology of the formulations. Double-sided adhesive tape was utilized to place the samples on an aluminum mount, which was sputtered with gold under vacuum and observed by scanning at an accelerated voltage of 15 KV.

### 3.10. In vitro Diffusion Studies

*In vitro* drug diffusion studies were conducted for all three batches of NSs (1:2, 1:4, and 1:8). The dialysis membrane method was employed to conduct the study. The membrane was immersed in pH 7.4 phosphate buffer for 24 h. Then, 150 mL of buffer was placed in the receptor compartment on a magnetic stirrer. NSs equivalent to 100 mg weight of the drug were placed on the membrane. The temperature was maintained at 37 ± 0.5 °C for 12 h while constantly stirring at 600 rpm. At regular intervals, 5 mL aliquots of the drug sample were taken out and replaced with the same volume of freshly prepared buffer solution. Utilizing a UV–visible spectrophotometer set at 239 nm, the drug analysis was carried out in triplicate. The released drug quantity was computed, and the percentage of drug released was plotted against time.

### 3.11. Preparation of Gel

The required amount of Carbopol 940 was allowed to soak in about 5 mL of water for 2 h. Badam gum was dissolved in a pre-measured quantity of propylene glycol. Once the solvent mixture had been transferred to a carbopol container, it was stirred for an additional 20 min. The resultant mixture was hydrated and left to swell for 60 min. The pH was adjusted to a range of 6.8–7.0 using 98% triethanolamine (TEA). The resultant mixture was then gently stirred using a spatula until a uniform gel formulation was achieved. Prior to the evaluation, a 24 h equilibration period at room temperature was provided for all samples [46,47]. Various polymer concentrations were utilized for the preparation of the gels are shown in Table 11.

### 3.12. Experimental Design

The relationships and interactions between dependent and independent variables can be studied systematically and scientifically using experimental designs. A randomized 3^2^ full factorial design, incorporating two factors across three distinct levels, was employed to methodically investigate the gel formulations. Nine experimental trials were conducted, encompassing all feasible combinations. The quantities of badam gum and carbopol were determined as independent variables, selected based on experiments conducted during the optimization of the excipient. These variables were altered at three levels: low, medium, and high. The levels of the factors under study were selected with the assumption that they were within the range of practicability and that their relative difference would be sufficient to measure an effect on the response. Spreadability and viscosity were considered as dependent variables (responses). The design and assessment of the statistical experimental design were carried out using the Design-Expert 10.0 software provided by Stat-Ease Inc., Minneapolis, Minnesota, USA. Different combinations of lumiracoxib NS-based topical gels using the 3^2^ full factorial design are depicted in Table 12.

### 3.13. Loading of Lumiracoxib Nanosponges into Topical Gels

An amount of NS equivalent to 200 mg of the drug was added to the 10 gm topical gel formulation. The gel was gently stirred to obtain a homogeneous product.

### 3.14. Characterization of Lumiracoxib NS Gel

#### 3.14.1. pH Determination

A digital pH meter (pH Tutor, Servewell Instruments Pvt. Ltd., Bengaluru, India) was utilized to determine the pH of the gel. An average of three readings were calculated [46,48].

#### 3.14.2. Homogeneity

The homogeneity of the formulated gels was evaluated by visual inspection after the gels were transferred to glass containers. The presence and appearance of any aggregates were checked closely [48].

#### 3.14.3. Spreadability Test

Weighed sample quantities (0.5 gm) were placed between two slides, and a weight of 500 gm was applied on the upper slide for about 5 min, where no more spreading was expected. The spread circle’s diameter, in centimeters, served as a standard for spreadability. Spreadability was analyzed in triplicate and was expressed in cm [47,48].

#### 3.14.4. Extrudability

With the help of a hardness tester, the extrudability test was conducted; 15 gm of sample gel was weighed in an aluminum tube. To adequately secure the tube, the plunger was adjusted. For 30 s, 1 kg/cm^2^ of pressure was applied, and the quantity of gel extruded was weighed. This procedure was repeated in triplicate [48,49].

#### 3.14.5. Rheological Studies

The rheological measurements were performed for all of the prepared gel formulations. All measurements were performed using cone-and-plate systems with a 1 mm gap and 50 mm diameter at 25 °C. At various shear speeds (rpm), the formulated gels’ rheological characteristics were assessed, and the viscosity was quantified in PaS [50].

#### 3.14.6. Drug Content

To a 100 mL volumetric flask, 100 mg of topical gel formulation was added, and methanol was used to make up the volume to 100 mL. Using a UV spectrophotometer, the absorbance was measured at 239 nm to estimate the drug concentration [51].

#### 3.14.7. *In vitro* Drug Diffusion Studies

An artificial cellophane membrane (Fisher Scientific Co., London, England) was used to permeate the lumiracoxib nanosponge-loaded nanogels. The maximum *in vitro* drug release was exhibited by formulation NS2 (1:4), which was therefore selected for this study. In the donor compartment, 0.5 gm of nanogel was placed. Then, pH 7.4 phosphate buffer was placed in a receptor medium and continuously stirred with the help of a magnetic bead. The temperature was maintained at a constant level of 37 ± 0.5 °C throughout the experiment to mimic the conditions of human skin. At 0.5, 1, 2, 6, 12, and 24 h, 5 mL aliquots were withdrawn and replaced with freshly prepared receptor solution. Spectrophotometric analysis was carried out for the withdrawn samples at 239 nm to calculate the amount of drug released. The drug release percentage was plotted against time, and the kinetics was examined using PCP-Disso version 2.0 software, BVDU’s Poona College of Pharmacy, Pune, India [52,53].

#### 3.14.8. Cytotoxicity Profile on L929 and HaCaT Cell Lines

Two cell lines were selected to evaluate the cytotoxic effects: L929 (mouse dermal fibroblasts) and HaCaT (human epidermal keratinocyte cells, a model for epidermal hyperproliferation in psoriasis). The cytotoxicity of the control, placebo, free LXB, and optimized gel formulation (FG10) was assessed using the MTT colorimetric assay. The cell lines were cultured in DMEM medium supplemented with 100 μg/mL streptomycin, 100 μg/mL penicillin, and 10% fetal bovine serum. The cell culture was maintained at 37 ± 0.5 °C in a humidified environment with 5% CO_2_. For the assay, 1 × 10^4^ cells were seeded in a 96-well plate and incubated for 24 h. After incubation, the medium was carefully removed, and the cells were treated with 100 µL of the test samples at different concentrations, from 0.1 to 100 µg/mL. Following 24 h of incubation, the cells were washed with phosphate buffer solution and treated with an MTT solution (5 mg/mL) for 4 h to allow formazan crystal formation. The crystals were then dissolved using dimethyl sulfoxide, and the plate was shaken for 20 min. Absorbance was measured at 570 nm using a microplate reader (Dynatech, Melville, NY, USA), and cell viability was calculated using the following formula:

$$\mathrm{Cell}\;\mathrm{Viability}\;(\%)\;=\;\frac{{OD}_{570}\;sample}{{OD}_{570}\;control\;}\times 100$$

where OD_570_ sample represents the OD of the treated cells, and OD_570_ control refers to the OD of the untreated control cells.

Additionally, the half-maximal inhibitory concentration (IC_50_) values, representing the drug concentration needed to inhibit cell viability by 50% compared to untreated controls, were calculated to assess the cytotoxic potency of the formulations [54,55].

#### 3.14.9. *In vivo* Studies [56,57,58]

- Animal grouping

Healthy BALB/c mice of both sexes, aged 8 to 11 weeks, were utilized for this study. The Institutional Animal Ethics Committee (IAEC) of JSS College of Pharmacy, JSS Academy of Higher Education and Research, Mysuru, approved the experimental procedures, including animal handling (Approval No. P144/2023). To induce psoriasis-like skin conditions, a daily topical application of 6 Imiquad^®^ (5% IMQ cream; Glenmark Pharmaceuticals Ltd., Mumbai, India) was applied to the shaved dorsal skin of the mice for 8 consecutive days. Table 13 outlines the animal groupings and their respective treatments.

b. Skin irritation studies

Acute skin irritation, potentially caused by the formulation or due to other factors, was assessed in the mice after shaving. The formulation was applied to the shaved skin, and the skin was monitored for signs of irritation, such as edema or erythema. These observations were made at 0, 24, 48, and 72 h post-application to evaluate any adverse reactions.

c. Skin inflammation studies

An ideal scoring system, modeled after the clinical Psoriasis Area and Severity Index (PASI), was designed to assess the severity of inflammation on the dorsal skin. Two key parameters—erythema (redness) and scaling—were independently evaluated and scored on a scale of 0 to 4 (0—none; 1—slight; 2—moderate; 3—marked; 4—very marked). This scoring system allowed for a standardized assessment of skin condition and inflammation severity.

#### 3.14.10. *Ex vivo* Permeation Studies

Skin samples were collected from healthy BALB/c mice post-euthanasia. The marketed gel and the optimized gel formulation (FG10) were applied to the shaved dorsal skin of the mice. The drug’s permeation through the skin was studied following a procedure similar to that used for *in vitro* drug diffusion experiments.

#### 3.14.11. Stability Studies

Stability studies were conducted under optimal temperature and relative humidity (RH) conditions to ascertain the shelf life of the product. For stability testing, the optimized formulation was selected. The optimized formulation was placed in capped bottles and kept in stability chambers for 30 days at a temperature of 25 ± 2 °C and 60 ± 5% RH. According to the ICH Q1A (R2) guidelines, samples were taken out on the 0th, 15th, and 30th days and spectrophotometrically measured at 239 nm for drug content analysis [42,48].

## 4. Conclusions

The developed nanosponge-based topical gel formulation demonstrated significant potential for the effective treatment of psoriatic arthritis. By utilizing a 1:4 polymer-to-crosslinker ratio, the nanosponges showed excellent drug encapsulation efficiency, enhanced solubility, and improved stability. The transition of lumiracoxib from a crystalline to an amorphous state, confirmed by DSC and XRD analyses, contributed to a remarkable 28–36-fold increase in solubility. The formulation achieved a sustained drug release of 86.12% over 24 h, with the Peppas model being the best fit for the release kinetics. Cytotoxicity studies on HaCaT cells confirmed the antipsoriatic activity of the formulation. With its favorable pH, viscosity, and spreadability, the topical gel not only offers localized, sustained release of lumiracoxib but also minimizes systemic side effects, making it a promising alternative to oral NSAID therapies. Clinically, this nanosponge-loaded topical gel offers significant advantages over conventional oral formulations. This optimized nanosponge-loaded gel represents a valuable advancement in the treatment of PsA, combining improved therapeutic efficacy with enhanced patient compliance.

## Figures and Tables

**Table 1:** Solubility studies for prepared nanosponge formulations.

Formulation	Solubility at pH 7.4 (µg/mL) (Mean ± SD *)
LXB	13.54 ± 0.06
NS1 (1:2)	363.74 ± 0.43
NS2 (1:4)	476.24 ± 1.09
NS3 (1:8)	423.74 ± 0.81

* SD = standard deviation, n = 3.

**Table 2:** The entrapment efficiency of prepared nanosponge formulations.

Sr. No.	Formulation	Entrapment Efficiency (%) (Mean ± SD *)
1	NS1 (1:2)	64.24 ± 0.050
2	NS2 (1:4)	72.38 ± 0.020
3	NS3 (1:8)	70.13 ± 0.015

* SD = standard deviation, n = 3.

**Table 3:** *In vitro* drug diffusion studies for formulations NS1 to NS3.

Time (h)	% Cumulative Drug Release (Mean ± SD *)		
	Nanosponge 1	Nanosponge 2	Nanosponge 3
0	0	0	0
0.5	16.34 ± 0.021	21.29 ± 0.025	19.18 ± 0.027
1	28.68 ± 0.018	34.49 ± 0.027	35.88 ± 0.023
2	40.12 ± 0.028	48.56 ± 0.023	53.33 ± 0.015
6	57.23 ± 0.025	63.42 ± 0.028	60.51 ± 0.030
12	69.42 ± 0.030	77.38 ± 0.018	72.14 ± 0.015
24	73.56 ± 0.026	86.12 ± 0.034	79.39 ± 0.024

* SD = standard deviation, n = 3.

**Table 4:** Layout of 3^2^ full factorial design and observed responses for LXB NS-based topical gels.

Formulation Code	A *	B *	R1 * (PaS) (Mean ± SD)	R2 * (cm) (Mean ± SD)
FG1	−1	1	0.58 ± 0.021	3.18 ± 0.025
FG2	−1	0	0.71 ± 0.034	4.55 ± 0.035
FG3	1	1	2.85 ± 0.041	10.51 ± 0.036
FG4	1	−1	2.23 ± 0.012	8.64 ± 0.026
FG5	−1	−1	1.15 ± 0.045	5.12 ± 0.041
FG6	1	0	2.82 ± 0.013	9.53 ± 0.025
FG7	0	0	1.69 ± 0.024	5.74 ± 0.033
FG8	0	1	2.19 ± 0.027	7.95 ± 0.031
FG9	0	−1	2.31 ± 0.054	9.26 ± 0.035

* A: carbopol (%); B: badam gum (%); R1: viscosity; R2: spreadability.

**Table 5:** Checkpoint analysis of optimized LXB nanosponge-based topical gel formulation (FG10).

Value	A	B	R1 (PaS)	R2 (cm)	Desirability
Predicted	0.27	0.45	1.13	6.96	0.981
Actual	0.27	0.45	1.15 ± 0.05	7.1 ± 0.03	
Relative error	-	-	0.02	0.14	

**Table 6:** *In vitro* release kinetics for topical gel formulation.

Formulation		Release Model			
		Zero-Order	Peppas	Higuchi	First-Order
NS 2	Ko (Slope)	3.1863	0.4562	-	−0.035
	R^2^	0.7190	0.9211	0.7743	0.9085

**Table 7:** Skin irritation studies.

Sr. No	Time (h)	Edema or Erythema
1	0	No sign
2	24	No sign
3	48	No sign
4	72	No sign

**Table 8:** Psoriasis Area and Severity Index (PASI) score observations.

Day	Group 1 *	Group 2 *	Group 3 *
0	0	4	4
2	0	4	3
4	0	4	2
6	0	4	2
7	0	4	1
13	0	4	0

* 0 indicates none; 1 indicates slight; 2 indicates moderate; 3 indicates marked; 4 indicates very marked.

**Table 9:** Stability studies for topical gel.

Stability Condition	Sampling Interval (days)	Physical Appearance	% Drug Content (Mean ± SD *)
25° ± 2 °C/60 ± 5% RH	0	No change	81.25 ± 0.0152
	15	No change	80.18 ± 0.0153
	30	No change	80.02 ± 0.0240

* The findings are expressed as the mean ± SD; n = 3.

**Table 10:** Formulation of nanosponges at three different ratios.

Sr. No.	Formulation	Polymer–Crosslinker Ratio (ß-Cyclodextrin–DMC)
1	Nanosponge 1 (NS1)	1:2
2	Nanosponge 2 (NS2)	1:4
3	Nanosponge 3 (NS3)	1:8

**Table 11:** Different polymer concentrations for the preparation of gels.

Sr. No.	Ingredients	Percentage (%)
1	Carbopol 940	0.2–0.4
2	Badam gum	0.4–0.6
3	Propylene glycol	0.5
4	Propyl paraben	0.02
5	Triethanolamine	Q.S
6	Water	Q.S upto 100 mL

**Table 12:** Composition of lumiracoxib NS-based topical gels using 3^2^ full factorial design.

Formulation Code	Concentration in Coded Factor Levels	
	A	B
FG1	−1	1
FG2	−1	0
FG3	1	1
FG4	1	−1
FG5	−1	−1
FG6	1	0
FG7	0	0
FG8	0	1
FG9	0	−1

Factors and their coded levels: for Factor A: carbopol (%): −1= 0.2, 0 = 0.3, 1 = 0.4; for Factor B: badam gum (%): −1 = 0.4, 0 = 0.5, 1 = 0.6.

**Table 13:** Animal groupings and their treatment.

Groups	Treatment
Group 1 (Control group; G1)	No treatment
Group 2 (Negative control group; G2)	Imiquad application and no treatment
Group 3 (Test group; G3)	Imiquad application and treatment with optimized gel formulation

## Data Availability

Data for the current research will be made available upon request to the corresponding author due to privacy and ethical restrictions.
